# Beautiful friendship: Social sharing of emotions improves subjective feelings and activates the neural reward circuitry

**DOI:** 10.1093/scan/nsu121

**Published:** 2014-10-08

**Authors:** Ullrich Wagner, Lisa Galli, Björn H. Schott, Andrew Wold, Job van der Schalk, Antony S. R. Manstead, Klaus Scherer, Henrik Walter

**Affiliations:** ^1^Universitätsmedizin Berlin, Department of Psychiatry and Psychotherapy, Division of Mind and Brain Research, 10117 Berlin, Germany, ^2^University of Münster, Department of Psychology, 48049 Münster, Germany, ^3^Berlin School of Mind and Brain, 10117 Berlin, Germany, ^4^Cardiff University, School of Psychology, Cardiff CF10 3AT, UK, and ^5^Swiss Center for Affective Sciences, 1202 Geneva, Switzerland

**Keywords:** social sharing, emotion regulation, reward, affiliation, fMRI

## Abstract

Humans have a strong tendency to affiliate with other people, especially in emotional situations. Here, we suggest that a critical mechanism underlying this tendency is that socially sharing emotional experiences is in itself perceived as hedonically positive and thereby contributes to the regulation of individual emotions. We investigated the effect of social sharing of emotions on subjective feelings and neural activity by having pairs of friends view emotional (negative and positive) and neutral pictures either alone or with the friend. While the two friends remained physically separated throughout the experiment—with one undergoing functional magnetic resonance imaging and the other performing the task in an adjacent room—they were made aware on a trial-by-trial basis whether they were seeing pictures simultaneously with their friend (shared) or alone (unshared). Ratings of subjective feelings were improved significantly when participants viewed emotional pictures together than alone, an effect that was accompanied by activity increase in ventral striatum and medial orbitofrontal cortex, two important components of the reward circuitry. Because these effects occurred without any communication or interaction between the friends, they point to an important proximate explanation for the basic human motivation to affiliate with others, particularly in emotional situations.

## INTRODUCTION

One of the most fundamental characteristics of human beings is their social nature. Humans have a strong motivation to form social bonds with conspecifics and to share their experiences with them (Baumeister and Leary, 1995; Rimé, 2009). Evolutionarily, the origin of this basic human ‘need to belong’ has been suggested to originate in the advantage of cooperative over individualistic work performance (Baumeister and Leary, 1995). Even when there is no specific task to perform, people tend to prefer to experience their environment together with peers rather than alone. Particularly in situations of enhanced emotional impact, people seek out the company of others (Schachter, 1959). By socially sharing their emotional experiences, individuals can apparently modify their subjective perception of these experiences in a positive manner. Accordingly, at the individual level, the human affiliative motivation might be linked to a fundamental hedonic mechanism related to the regulation of individual emotions (Rimé, 2009). To illustrate, take the simple everyday example of a visit to the cinema: When people go to the cinema to watch a film, they rarely do this alone, but in most cases go together with a partner or friend. Apart from expecting to be emotionally moved by the film itself, they anticipate a positive subjective impact of sharing this emotional experience with a peer, even though both of them are only passively watching an event and there are only minimal opportunities to talk to each other during the viewing. Do people appreciate the company of others because their mere knowledge of the presence of a peer who shares the same emotional experience is subjectively rewarding?

Here, we tested this idea with subjective ratings and functional magnetic resonance imaging (fMRI) data from participants who viewed emotional pictures while a friend either watched (shared emotional experience) or did not watch (unshared emotional experience) the same stimuli simultaneously. We predicted that socially shared as opposed to unshared emotional experiences would result behaviorally in more positive affective ratings and neurobiologically in a concomitant activation of the dopaminergic reward sytem in the brain encompassing the ventral striatum (VS) and the ventromedial prefrontal/orbitofrontal cortex (VMPFC/OFC) (Schultz, 2006; Peters and Büchel, 2010; Grabenhorst and Rolls, 2011). If confirmed, this would reveal an important proximate mechanism underlying the inherently social nature of human beings.

Although direct evidence is still missing, several strands of evidence suggest that socially sharing an emotional event does indeed have a subjectively rewarding character. Research on facial displays has shown that smiling increases and that sad expressions are reduced in the presence of friends (Fridlund, 1991; Hess *et al.*, 1995; Jakobs *et al.*, 2001). In these studies, participants watched emotional film scenes alone, together with a friend, or in the knowledge that the friend was watching simultaneously in another room. Watching together increased the frequency of smiling and reduced the frequency of sad expressions. However, in view of discussions on the interpretation of facial expressions as involuntary communicative signals rather than reflections of subjective emotional states these data do not necessarily imply genuinely changed individual feelings (Fridlund, 1991; Parkinson, 2005).

Regarding neuroimaging, a variety of recent fMRI studies have demonstrated the social sensitivity of the human reward system. For example, the VS shows activation toward both monetary and social reward (Izuma *et al.*, 2008; Rademacher *et al.*, 2010), and winning for a friend in a gambling game is experienced to be as rewarding as winning for oneself (Braams *et al.*, 2014). Similarly, interactions with friends, even when simulated in an fMRI environment, can be rewarding and activate reward-related brain areas including the striatum, as well as the VMPFC (Guroglu *et al.*, 2008). Finally, joint attention, established by looking at the same object as an interaction partner, is experienced as more pleasant than looking alone and recruits reward-related brain networks, including the medial OFC and VS (Schilbach *et al.*, 2010). Together, these studies clearly show that the human reward system reacts to various manipulations of social information processing. However, to date no study has investigated a social role of the human reward system in basic processing of externally induced emotions, independent of decisions in gambling tasks or direct social signals from a partner’s face. In the present study, we aimed to investigate the involvement of the human reward system in individual emotion modification by the mere belief that an affective situation is socially shared. Our study thereby critically extends existing paradigms and addresses a new and thus far neglected functional aspect of this system, namely, as the intrinsic motivational basis of human affiliative tendencies even in the absence of external social cues.

A primarily negative or positive emotional experience, which could be socially shared or not, was elicited by a standardized set of photographs with emotional content (Lang *et al.*, 2005). Similar to the procedures in the facial expression studies mentioned above (Fridlund, 1991; Hess *et al.*, 1995; Jakobs *et al.*, 2001), the study was performed with pairs of friends, and for each picture, the social context manipulation was reduced to the mere information that the friend, who was sitting in an adjacent room, either would or would not simultaneously view the same picture. Thus, in the absence of any online social information exchange or decision-making, the social dimension of the situation was defined solely by the subjective interpretation of the externally generated emotional experience as one that was or was not shared with a friend.

**Fig. 1:**
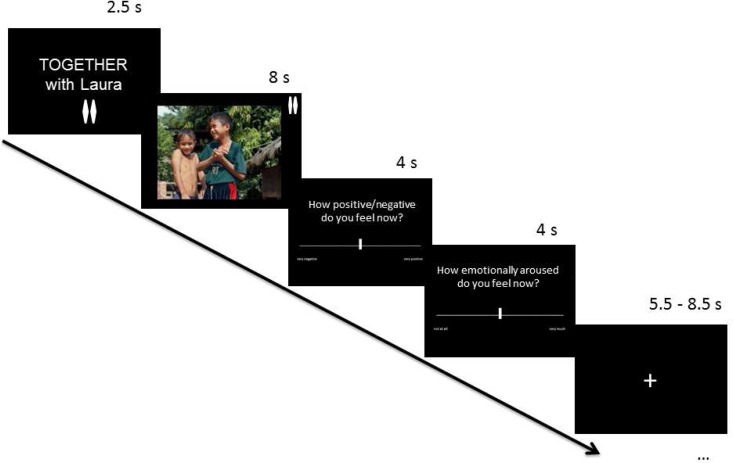
Trial structure. Each trial started with a cue announcing whether the subsequent picture would be watched together with the friend (shared experience, as in this example trial) or alone (unshared experience). The picture, whose content could be emotionally positive (as in this example), emotionally negative or neutral, was then presented, followed by subjective emotion ratings of valence (very negative to very positive) and arousal (very low to very high). To ensure that participants were always aware of the experimental condition, small symbols for the shared (two rhombs) *vs* unshared (one rhomb) condition were also visible during picture viewing.

Before a picture was presented, an announcement indicated whether it would also be seen by the friend (Figure 1). When the picture was to be seen only by the participant, the friend would perform an unrelated attention task. After each picture, participants rated their current emotional state on rating scales of valence (negative to positive) and arousal (low to high). According to our hypothesis that social sharing of emotions per se is hedonically rewarding, we expected a positive shift of valence ratings in shared (relative to unshared) emotional picture processing, irrespective of the primary picture-induced emotional state (negative or positive), and that this effect would be accompanied by an activation of the VS and VMPFC/OFC. Although our hypothesis pertained primarily to the sharing of emotional (negative or positive) experiences, we also included neutral pictures, which allowed us to examine the extent to which any social sharing effects were specific to inherently emotional experiences.

## METHODS

### Participants

Sixty female volunteers (30 pairs of friends) completed the experiment after giving informed consent. Magnetic resonance imaging (MRI) data were obtained from 30 of them. They were recruited from the Charité Universitätsmedizin Berlin and the Free University Berlin, and through social media advertisements. The study was approved by the ethics committee of the Charité Universitätsmedizin Berlin, Campus Mitte. Participants were aged between 20 and 33 years (*M* = 24.76, SEM = 0.364) and fit common inclusion criteria for fMRI research. Each participant received 25 or 30 Euros depending on the duration of the session. A control questionnaire confirmed that the two participants in each pair regarded each other as close friends [on a scale from 0 (not true at all) to 10 (entirely true), the item ‘How close is this friend to you?’ was rated with *M* = 8.00, s.d. = 1.775; the item ‘How important is this friendship for your life?’ was rated with *M* = 8.667, s.d. = 1.348]. Two participants were excluded from fMRI analysis owing to technical error in the acquisition of brain images. Two further participants reported developing a headache in the scanner and were therefore excluded from both behavioral and fMRI data analyses. Thus, behavioral analysis included *n* = 58 participants, and fMRI analyses *n* = 26 participants.

### Task and experimental design

Participants rated their emotions in response to negative, positive and neutral pictures from the International Affective Picture System, from which pictures were chosen on the basis of their valence ratings by a female standardization sample (Lang *et al.*, 2005). Fifty-four pictures were selected for negative (e.g. car accidents), positive (e.g. food) and neutral (e.g. household objects) content, respectively. Emotional pictures were chosen to be as extreme as possible while keeping positive and negative pictures on average equally extreme, by balancing for the distance from the scale midpoint. Neutral pictures clustered around the midpoint of the scale (details of the stimulus sets are listed in Supplementary Table S1). Assignment of picture sets to the shared *vs* unshared condition was counterbalanced across subjects. To further reduce possible biases in within-subjects comparisons, we applied a within-subjects correction procedure for the calculation of the behavioral sharing effect (see below).

To exclude possible confounds by subtle effects elicted by social *vs* non-social content of the pictures themselves, the sociality of picture content was matched within each picture category in each picture set. Specifically, within negative, positive and neutral pictures, one-third of the pictures showed humans with faces visible, humans without faces visible and inanimate motives, respectively, according to the content classification by Colden *et al.*(2008). Control analyses including picture content as an additional analysis of variance (ANOVA) factor showed that this factor did not interact with the sharing effect in valence ratings [*F*(2,112) = 0.931, *P* = 0.397] and was therefore not considered in further analyses. E-Prime Software (v2.0, Psychology Software Tools, Inc) was used to program and control stimulus presentation. Stimulus presentation was pseudo-randomized such that no more than two pictures from the same valence category (negative, positive and neutral) were presented consecutively. Similarly, no more than two consecutive trials had the same sharing condition (‘alone’ *vs* ‘together’).

Each trial (see Figure 1) consisted of a 2500 ms written announcement of the sharing condition (‘TOGETHER with [name of friend]’or ‘ALONE’), followed by a stimulus picture presented for 8000 ms. The sharing condition was further indicated by one (unshared) *vs* two (shared) small elongated rhombs in the top right corner of each picture, so that even in phases of attenuated attention participants could not forget whether the picture was viewed alone or together with the friend. After the picture had disappeared from the screen, participants rated their subjective feelings in terms of valence and arousal. For each of the two ratings (valence and arousal), participants had 4 s to log their rating by moving a cursor along a scale ranging from ‘very negative’ to ‘very positive’ for valence and from ‘very low’ to ‘very high’ for arousal. The order of the two ratings was randomized for each trial. While the rating scale consisted of an unmarked line, the cursor moved in small steps (not continuously), resulting in a 13-point scale. Each trial concluded with a fixation cross presented for an average of 7000 ms (jittered; range = 5500–8500 ms).

The experiment was completed inside the MRI scanner by one participant and in an adjacent room by the other. During ‘together’ trials, both participants, while in separate rooms, viewed the same picture at the same time. During ‘alone’ trials, the participant supposedly viewed a picture alone, with the friend completing at the same time an unrelated attention task (responding by button press to five single-digit numbers shown consecutively for 2 s each, deciding whether each number was bigger or smaller than five). In fact, the two participants completed exactly the same order of trials and stimuli. However, the unrelated attention task trials included served to make the setup of shared *vs* alone trials plausible. The experiment consisted of three runs of 36 trials of interest plus 5 unrelated attention task trials each, resulting in an overall duration of ∼50 min. Before each run, participants were reminded by the experimenter that their friend was completing the experiment simultaneously in the other room.

In the scanner, stimuli were projected onto a screen behind the head of the participant, which the participant could view via a mirror system mounted on the head coil. In the adjacent room, the experiment was simultaneously run on a laptop for the friend. To log their responses, participants used three predefined keys on a four-button box (in fMRI scanner) or on the laptop keyboard (in the adjacent room). Two of the three keys enabled participants to move a cursor indicating the position on a rating scale to the left or to the right, the third key served to confirm the current position of the cursor on the scale as the final rating response.

A practice phase before the main experiment served to familiarize participants with the trial structure and the general procedure, and to clarify that they would be completing the experiment simultaneously, with some pictures viewed together and others alone. For this purpose, the two friends performed this practice phase side by side on two directly adjoining computers, so that each of the friends could see simultaneously their own and the friend’s computer monitor. The practice phase was constructed in such a way that the participants could see that the ‘alone’ trials were different from the ‘together’ trials, in line with the experimental setup.

### Behavioral data analysis

The primary behavioral variable of interest was the sharing effect in participants’ subjective ratings of valence in response to the pictures, i.e. the difference between ratings in the shared and the unshared conditions (with positive values indicating a shift toward more positive feelings in the shared condition). The social sharing effect was tested against zero to specify whether there was an effect of social sharing overall or in the emotional or neutral conditions separately. Comparisons of sharing effects between conditions were performed by a one-way repeated measures ANOVA and (in the case of comparisons involving two conditions) paired *t*-tests.

In addition to balancing the assignment of picture sets to the shared *vs* unshared condition across subjects, we introduced a within-subjects correction procedure: for each participant and each category, the mean difference in ratings between shared and unshared pictures of the standardization sample (Lang et al., 2005, adapted to scale range of 0 to 12) was subtracted from participant’s own mean difference in ratings between shared and unshared pictures. Although these differences in standardization sample ratings were small owing to our picture matching procedure, they were naturally still slightly different from zero individually and could thus bias the likewise subtle sharing effect to some degree without this correction. Although analyses using the uncorrected raw sharing effect instead of the corrected sharing effect yielded essentially the same results (see Supplementary Table S2), we report throughout this article the corrected values as the bias-corrected estimate of the actual sharing effect. However, raw data are reported when absolute ratings rather than differences between shared and unshared conditions are presented.

Because the sharing effects in emotion ratings did not differ between participants inside the fMRI scanner and those outside (*P* > 0.20, for all effects involving group inside *vs* group outside scanner), the group factor was not considered in further analyses.

### fMRI data acquisition and analysis

MRI data were acquired on a Siemens Tim Trio 3T MRI scanner at the Berlin Center for Advanced Neuroimaging, using a 12-channel head coil. Functional images were acquired in descending order with a T2*-sensitive EPI sequence in 32 contiguous 3 mm axial slices in an oblique orientation of −18° to optimize for signal detection in ventral prefrontal areas (TR/TE/flip angle = 2000 ms/30 ms/78°, FOV = 192 mm × 192 mm, slice thickness 3.0 mm). Whole-brain anatomical images were acquired after the end of the experimental task with an MPRAGE T1-weighted sequence (TR/TE/flip angle = 1900 ms/2.52 ms/9°, FOV = 256 mm × 256 mm, slice thickness 1 mm).

The images were analysed using SPM 8 (Wellcome Department of Imaging Neuroscience, London, UK; http://www.fil.ion.ucl.ac.uk/spm). Motion correction was performed by realignment to the first image. After motion correction, EPIs were unwarped based on fieldmaps acquired in the end of the scanning session and corrected for acquisition delay, with the 17th (middle) slice as reference. EPIs were then normalized onto a standard MNI EPI template and smoothed with a Gaussian kernel of 8 mm FWHM. A high-pass filter with a cutoff period of 128 s was applied to the data to remove drifts within sessions.

Statistical analysis was performed using the two-stage mixed-effects General Linear Model (GLM) approach implemented in SPM8. At the first stage (single subjects), box-car functions at stimulus onset for the different event types were convolved with SPM8’s hemodynamic response function to form covariates of a GLM. The GLM included six regressors representing the experimental conditions (shared/unshared × positive/negative/neutral) during picture phase (8 s). Four additional regressors—two regressors representing the instruction phase (shared/unshared; 2.5 s), one regressor for unrelated attention task trials (2.5 s instruction + 8 s task) and one regressor for the rating phase (8 s)—were modeled as covariates of no interest. Furthermore, the six movement parameters obtained from realignment were included as regressors of no interest to account for residual movement variance. Fixation time served as baseline, and a single constant represented the mean over scans.

At the second stage of the model, individual *t*-contrast maps were submitted to a second-level random effects analysis, applying a full-factorial within-subjects ANOVA model (independence not assumed, equal variance assumed) with two factors (sharing: 2 levels/emotion category: 3 levels). Planned comparisons were computed as linear contrasts of the parameter estimates within this model.

The significance threshold at voxel level was set to *P* < 0.001 uncorrected and a minimal cluster size of 10 voxels. Cluster-level thresholding for whole-brain analyses was performed with AFNI AlphaSim (Ward, 2000) implemented in SPM8 (whole-brain corrected *P* < 0.05). Regions of interest (ROI) analyses were performed based on our a priori hypotheses of involvement of VS and OFC in the sharing of emotions. For the OFC a probabilistic literature-based ROI was created using a previously described algorithm (Schubert *et al.*, 2008; Zweynert *et al.*, 2011). To this end, VS and OFC peak coordinates were selected from previous fMRI studies (for VS: Zweynert *et al.*, 2011; for OFC: Schott *et al.*, unpublished data, see also Supplementary Table S4). These ROIs represent the two principal regions of reward processing, specifically positive reward, as identified in a comprehensive meta-analysis by Liu *et al.* (2011).

### Assessment of social motives

To examine directly the role of friendship-related social motives in the observed rewarding effects of social sharing, we calculated correlations with self-report measures (obtained after scanning) on social motives, which were assessed by a modified version of a questionnaire by Jakobs *et al.* (2001). The questionnaire includes four items relating to awareness of and thoughts about the friend (‘I was thinking of my friend’; ‘I was aware of my friend’s presence’; ‘I was wondering how she would find the pictures’; and ‘I was wondering how she was feeling while seeing the pictures’) and three items relating to intentions to communicate with the friend (‘I wanted to tell her what I thought about the pictures’; ‘I had the urge to talk about the pictures with her’; and ‘I imagined how we would talk about the pictures after the experiment’). Thus, two different, although related, aspects of social motives are assessed, which can be referred to as ‘awareness of the friend’ and ‘communicative motives’, respectively. Ratings were made on a 7-point Likert scale ranging from 0 (not true at all) to 6 (entirely true). For the purpose of a rough manipulation check, the questions were asked for both the shared and the unshared conditions separately, and within these conditions for the emotional *vs* neutral picture categories separately (e.g. for shared/negative: ‘When seeing negative pictures together with my friend I was aware of my friend’s presence’). These differential answers confirmed stronger social motives (awareness and communication) for shared compared with unshared experiences (*P* < 0.001) and for emotional compared with neutral experiences (*P* < 0.001), as expected.

To test whether the effects of social sharing in VS and/or OFC are directly related to indiviual social motives, correlations were calculated between the social motives ratings and the extent of the observed sharing effects in brain activation. We used the ratings from shared conditions to calculate the reported correlations with brain activations because these conditions are inherently most pertinent to social motives.

## RESULTS

### Social sharing effects on subjective emotion ratings

**Fig. 2:**
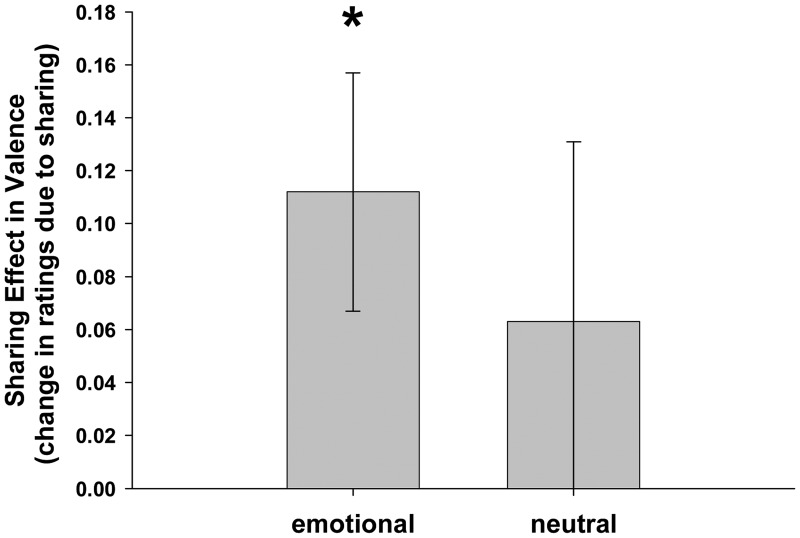
Sharing effect in valence ratings (mean differences between shared and unshared condition, corrected for standard ratings) for emotional and neutral pictures (*n* = 58). Social sharing increases subjectively perceived valence, indicated by overall positive values, especially when pictures with emotional content are experienced (*P < 0.05, for difference from zero). Bar for emotional pictures combines sharing effects for positive and negative pictures, which do not differ from each other. Error bars represent ± 1 SEM.

As predicted, participants reported more positive feelings when viewing emotional pictures with a friend than when viewing alone, as indicated by a positive sharing effect on valence ratings for emotional (negative or positive) pictures [*M* = 0.112, SEM = 0.045, *t*(57) = 2.469, *P* = .017], with no difference between the two emotion categories [negative: *M* = 0.088, SEM = 0.057, *vs* positive: *M* = 0.136, SEM = 0.074; *t*(57) = 0.502, *P* = .618]. Regarding neutral pictures, there was a positive but nonsigificant sharing effect [*M* = 0.064, SEM = 0.068; *t*(57) = 0.933, *P* = 0.355]. There was no significant difference when the neutral pictures were directly compared with the emotional picture categories [*t*(57) = 0.578, *P* = 0.565], and the sharing effect remained significant when neutral pictures were included [*M* = 0.0956, SEM = 0.0373; *t*(57) = 2.559, *P* = 0.013]. Thus, the positive effect of social sharing on the subjective level extends at least to some degree to neutral stimuli (Figure 2).

Social sharing had no effect on arousal ratings (see Supplementary Results and Supplementary Table S2 for all raw data on subjective ratings of valence and arousal).

### fMRI results

A comparison of brain responses to shared *vs* unshared emotional pictures (shared_emo > unshared_emo; Table 1) revealed activation in the right VS and the left OFC (*P* < 0.05, small-volume family-wise error (FWE)-corrected for the respective ROIs). Although peak activations were right-sided for the VS and left-sided for the OFC, control analyses showed that positive (but subthreshold) activations were also present on corresponding contralateral regions, and there was no statistically significant lateralization effect. An exploratory analysis of additional sharing-related differences in brain activity beyond these ROIs revealed increased activation of the left dorsolateral prefrontal cortex (DLPFC) and bilateral precuneus (*P* < 0.05, whole-brain corrected). Unlike emotional pictures, neutral pictures elicited no effect of social sharing (shared_neu > unshared_neu: no suprathreshold brain activation at *P* < 0.001, uncorrected), and the above-mentioned activations for the sharing of emotions were attenuated to a subthreshold level when emotional and neutral pictures were combined (shared > unshared). Increased activation for unshared as compared with shared trials was found in the lingual gyrus, for both emotional and neutral stimuli (Supplementary Table S3).

**Table 1:** Regional activation during picture viewing

Brain region	Hem.	Brodmann area	Coordinates peak voxel			*T*-value	Cluster size
			x	y	z		
*Shared_emo > unshared_emo*							
VS^a^	R		9	5	−2	4.09	19
VMPFC/OFC^a^	L	11	−9	32	−11	4.4	61
Anterior cingulate	L	32	−18	32	19	4.17	14
	R	32	15	32	19	4.07	19
Frontopolar cortex	L	10	−3	59	−5	3.87	18
	L	10	−18	59	22	3.58	18
DLPFC^b^	L	8	−33	26	43	4.22	280
	L	9	−18	35	49	4.15	
	L	9	−24	35	40	3.85	
Precuneus^b^	L/R	7	0	−55	31	4.31	274
	R	31	6	−49	37	4.09	
	L	7	−3	−61	49	3.91	
Inferior parietal lobule	L	40	−51	−61	40	3.52	40
Cerebellum	R		33	−82	−32	3.56	23
							
*Shared_emo > unshared_emo (masked with interaction sharing × emotion)*							
VS^a^	R		9	5	−2	4.09	13
VMPFC/OFC^a^	L	11	−9	32	−11	4.4	55
Anterior cingulate	L	32	−18	32	19	4.17	12
	R	32	15	32	19	4.07	18
DLPFC^b^	L	8	−33	26	43	4.22	161
	L	9	−18	35	49	4.15	
Precuneus	R	31	6	−49	37	4.09	13
	L	7	−6	−58	43	3.82	11
Inferior parietal lobule	L	40	−51	−61	40	3.52	17
							
(shared_pos > unshared_pos) > (shared_neg > unshared_neg)							
no suprathreshold voxels at *P* < 0.001 uncorrected, k > 10							
(*shared_neg > unshared_neg*) > (*shared_pos > unshared_pos*)							
no suprathreshold voxels at *P* < 0.001 uncorrected, k > 10							
*shared_neu > unshared_neu*							
no suprathreshold voxels at *P* < 0.001 uncorrected, k > 10							

*Notes:* Peak voxels in MNI space, *P* < 0.001 uncorrected, k > 10.

^a^Significant after small-volume FWE voxel-level correction within a priori ROI.

^b^Significant after whole-brain cluster-level correction, *P* < 0.05.

**Fig. 3:**
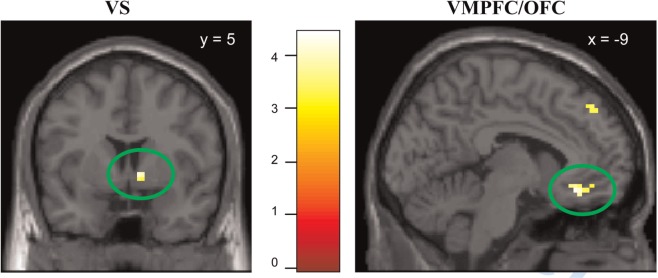
Brain activation in ROIs related to social sharing of emotions (contrast shared_emo > unshared_emo, masked with the interaction sharing × emotion), shown at *P* < 0.001, uncorrected. Left: Ventral striatum Right: Ventromedial prefrontal cortex/orbitofrontal cortex. Activations are significant at *P* < 0.05, FWE corrected within ROIs.

To further assess whether the social sharing effect was specific to the emotional categories, the T contrast of sharing for emotional pictures (shared_emo > unshared_emo) was inclusively masked with the F contrast of the sharing by category interaction contrast (shared/unshared × negative/positive/neutral). Activations within the reward circuitry (VS, OFC) remained significant (*P* < 0.05, FWE-corrected for ROI volumes) after masking, indicating specificity of sharing effects in these regions to emotional stimuli (Table 1, Figure 3). The sharing-related activation differences in DLPFC and precuneus were also located within the mask, although whole-brain cluster-level correction remained significant after masking only for the DLPFC (Table 1).

We found no evidence for a further modulation of the sharing effect for emotional trials by positive *vs* negative valence, as a direct comparison of the sharing effects for negative and positive stimuli (shared_neg > unshared_neg) > (shared_pos > unshared_pos) and (shared_pos > unshared_pos) > (shared_neg > unshared_neg) elicited no suprathreshold activation at *P* < 0.001, uncorrected (Table 1), although separate interaction contrasts for the comparison of the sharing effect for negative and positive stimuli with the neutral condition, i.e. (shared_neg > unshared_neg) > (shared_neu > unshared_neu) and (shared_pos > unshared_pos) > (shared_neu > unshared_neu), showed suprathreshold activation in the ROIs only in the former case.

For a more fine-grained analysis of the pattern of activation in both VS and OFC, we extracted the actual parameter estimates (beta values) for brain activation separately for all experimental conditions for statistical comparisons. This analysis confirmed (i) that the pattern indeed differed between emotional and neutral conditions (with higher values in the shared than the unshared condition for emotional (negative and positive) pictures, but lower values in the shared than the unshared condition for neutral pictures (means ± SEM for shared *vs* unshared conditions in VS: emotional pictures 0.165 ± 0.033 *vs* 0.094 ± 0.030, neutral pictures 0.089 ± 0.036 *vs* 0.110 ± 0.034; *F*(1,25) = 11.3, *P* = 0.003, for interaction; in OFC: emotional pictures 0.043 ± 0.013 *vs* − 0.008 ± 0.015, neutral pictures − 0.012 ± 0.036 *vs* 0.015 ± 0.014, *F*(1,25) = 18.9, *P* = 0.0001 for interaction), and (ii) that within emotional pictures there was no statistical difference between the sharing effect for negative and positive pictures, although the effect was numerically stronger for negative than positive stimuli (means ± SEM for shared *vs* unshared conditions in VS: negative pictures 0.210 ± 0.040 *vs* 0.100 ± 0.029, positive pictures 0.121 ± 0.035 *vs* 0.089 ± 0.038, *F*(1,25) = 2.50, *P* = 0.13, for interaction; in OFC: negative pictures 0.039 ± 0.017 *vs* −0.027 ± 0.016, positive pictures 0.046 ± 0.017 *vs* 0.012 ± 0.018, *F*(1,25) = 1.52, *P* = 0.23 for interaction).

We also extracted the parameter estimates for the regressors relating to the instruction phase preceding the presentation of the actual picture in each trial, to explore whether activation in VS or OFC would be already induced anticipatorily by the announcement that the subsequent picture would be seen together with the friend rather than alone. Such an anticipatory effect was not found, as brain activation estimates did not differ significantly between ‘TOGETHER’ *vs* ‘ALONE’ instruction slides, with in fact even lower values in the ‘TOGETHER’ than in the ‘ALONE’ condition (means ± SEM for ‘TOGETHER’ *vs* ‘ALONE’ conditions in VS: 0.004 ± 0.058 *vs* 0.041 ± 0.060, *t*(25) = −0.91, *P* = 0.37; in OFC: −0.019 ± 0.035 *vs* −0.028 ± 0.035, *t*(25) = −0.35, *P* = 0.73).

Irrespective of sharing conditions, strong main effects of emotion (emotional > neutral) occurred during picture viewing, mainly in bilateral amygdala, in bilateral medial temporal gyrus and in the precuneus/posterior cingulate (see Supplementary Table S3, for detailed brain activations in contrasts emotional > neutral, negative > positive and negative > positive).

### Correlations of social motives with sharing effects in ROIs

Awareness of the friend correlated with right VS activation during presentation of emotional pictures (*r* = 0.42, *P* = 0.03) and at the same time predicted the extent of the positive sharing effect in valence ratings (*r* = 0.41, *P* = 0.03). The correlations between communicative motives and sharing effects were also positive but did not reach significance. Neither awareness of the friend nor communicative motives were correlated with OFC activation during picture viewing (all *P* values > 0.67). There was also no significant direct correlation between the sharing effect in valence ratings and sharing-induced brain activation increase in the VS or OFC.

## DISCUSSION

Humans, as inherently social beings, show a strong inclination to affiliate with each other and in particular to share their emotions with peers (Baumeister and Leary, 1995; Rimé, 2009). This sharing is normally accompanied by verbal and non-verbal communication signals between partners. To unambiguously reveal effects of social sharing per se, independent of (a) any associated direct social interaction or communication and (b) cognitive processing related to specific reward-associated tasks, we investigated how the mere fact that a friend is simultaneously sharing the same emotional experience (induced by negative and positive pictures) changed individual affective states with regard to subjective feelings and brain activation. Participants reported significantly more positive affect when viewing emotional pictures together with a friend than when viewing them alone, and this effect was accompanied by activation of reward-related brain areas, i.e. VS and OFC. Notably, this effect was independent of whether the emotional state elicited by the pictures was negative or positive. Our results suggest a psychological and neurobiological mechanism underlying the human tendency to seek the company of peers in emotional situations. Psychologically, emotional episodes are rendered more pleasant when they are shared, and neurobiologically, this sharing activates the neural reward system.

The behavioral data are consistent with previous findings of sharing effects on facial expressions, showing enhanced positive and reduced negative facial expressions when participants watched emotional films together with a friend, even if the friend watched it in a separate room (Fridlund, 1991; Hess *et al.*, 1995; Jakobs *et al.*, 2001). In contrast to those studies, however, our findings also demonstrate a substantial positive sharing effect on subjective feelings. Automatically guided facial expressions are likely to be more sensitive to subtle manipulations of social context than explicit subjective ratings. Thus, the fact that we did find a sharing effect in subjective ratings may result from the more powerful within-subjects design used here, with a great number of stimuli shown, that was able to detect even a relatively small effect. With regard to debates on whether effects of emotion sharing would be the same or different for positive and negative emotional situations (Fischer *et al.*, 2003; Rimé, 2009), the present results indicate that sharing renders all emotional experiences more pleasant, whether they are intrinsically negative or positive. Moreover, unlike the above-mentioned studies on sharing that exclusively showed emotional (positive or negative) film material to be watched either together or alone, we also included neutral stimuli here and found, at least at the psychological level, that the positive effect of sharing to some degree extended to neutral pictures (where no primary emotion was elicited that could be shared), although it was numerically less pronounced than when the pictures did elicit emotions.

These findings confirm and extend existing accounts of social emotion regulation (Rimé, 2009; Gable and Reis, 2010; Zaki and Williams, 2013). Specifically, the idea of these accounts that social sharing positively affects individual emotions is clearly supported, and as in the previous literature on emotion sharing, the beneficial effect was found similarly for both negative and positive emotions (Rimé 2009; Gable and Reis, 2010; Zaki and Williams, 2013). However, this previous research primarily refers to explicit sharing, i.e. the act of communicating with another person about an emotional event after the fact (see Echterhoff *et al.*, 2009, for different semantic aspects of the term ‘sharing’ in social psychology). The present results extend this line of research by showing that the mere knowledge of the presence of a peer improves subjective feelings, in the absence of any direct contact, communication or interaction—an implicit effect that is present already at the time when the emotion is elicited, possibly as a result of an imagined contact with the friend.

In line with our hypotheses, the behavioral effect of sharing was complemented at the neural level by increased activation of the reward system in the VS and medial OFC during shared (compared with unshared) trials. A variety of previous studies has already shown these areas of the human reward system to be sensitive to social information processing. For example, activity in the VS has been consistently linked with the representation of both primary rewards and social rewards (Izuma *et al.*, 2008; Rademacher *et al.*, 2010). Similarly, sharing monetary gains from a gambling game activates VS more when sharing with friends than with other peers or a computer (Fareri *et al.*, 2012). Even more pertinent to the present study are studies that used minimal social interaction manipulations. For example, Schilbach *et al.* (2010) showed in an eye-gaze following task that joint attention is experienced as pleasant and activates the VS and OFC. Other studies have shown activation of the VS by simulated interactions with friends (Guroglu *et al.*, 2008), and activation of the medial OFC by experiencing emotional synchrony, i.e. watching an emotional facial expression congruent with one’s own current emotions (Kühn *et al.*, 2011). The present study critically extends these findings by further minimizing the social situation to a symbolic level, without facial signals from the interaction partner. In this way, we could interpret the effects as resulting from an intrinsic social motivation rather than an effect of communicative signals or actual interactions. Interestingly, even this minimal social processing had the same rewarding effect for negative and positive emotional states, like the emotional synchrony between two persons observed by Kühn *et al.* (2011). The fact that such responses can be triggered by symbolic indicators of social sharing demonstrates the intrinsic value we humans find in connecting with other people (Baumeister and Leary, 1995; Fiske, 2004). Positive correlations of the extent of the sharing effects with the extent of the social motives reported by the subjects additionally support the view of social motivational factors as the source of the effects, but also show that individual differences have to be considered here.

In addition to our predefined ROIs, we observed increased activation of the precuneus and the DLPFC and the precuneus for shared over unshared trials with whole-brain correction. In the present context, recruitment of these structures is likely related to the awareness of and thoughts about the friend during picture viewing. Notably, the DLPFC is also known as one of the primary brain areas involved in emotion regulation (Ochsner and Gross, 2005; Walter *et al.*, 2009). Thus, activity in this region may also reflect an emotion-regulating effect of social sharing, in this case even in the absence of any direct instructions to regulate emotions, extending previous findings concerning the role of the DLPFC in intentional individual emotion regulation.

While we did not find differential sharing effects for negative and positive emotional stimuli, either behaviorally or neurally, an additional research question was to which extent sharing effects predicted for emotional events would generalize to neutral events. The consequences of sharing neutral experiences seem harder to predict and have not yet been considered in previous research. It seems plausible that sharing effects would be conditional on the presence of an emotion that affords social buffering (of negative feelings) or enhancement (of positive feelings). On this account, neutral situations would remain unaffected because there is neither a need for comfort nor an opportunity to share enjoyment. Our data indicate that sharing neutral experiences is indeed less rewarding than sharing emotional experiences, at least at the neural level. Nevertheless, at the behavioral level, results indicated that subjective valence was also more positive to some degree for shared, rather than unshared, neutral stimuli. These partly divergent results at the neural and psychological levels may to some degree reflect methodological issues, possibly arising from transfer effects between trials in our within-subjects design, which might be more evident at the behavioral level than in the slowly reacting hemodynamic response in fMRI.

We deliberately chose to recruit close friends because these are the persons with whom individuals tend to prefer to share emotions in everyday life. It is possible that our finding of a hedonic gain induced by sharing of emotions in general, i.e. of both negative and positive experiences, is specific to friendship or close relationships. One could speculate of at least some functional commonalities between the sharing of positive and negative emotions on the basis of these findings. For example, in both cases, the feeling of connectedness to the friend may be enhanced, thereby further strengthening the relationship. Also, the basal ganglia, to which the VS belongs, are known as a brain structure critically involved in the initiation of voluntary motor activity (e.g. Nelson and Kreitzer, 2014). Thus, for both negative and positive emotional contexts, the activation of the VS, in addition to the effect on subjective feelings, may also indicate enhanced preparedness for action, in this case most likely joint action. Such secondary consequences could induce additional long-term effects far beyond the initial hedonic gain experienced within the initial situation.

We are not arguing that close friendship is absolutely necessary to elicit the sharing effects observed here, but the situation could be different if the two persons who share an emotional experience are not friends, but strangers or even opponents. It is likely that sharing can even influence individual feelings negatively in such cases, and that the sharing effect might be different in positive and negative emotional situations, depending on the context and individual factors. Previous researchers have already noted that sharing effects can depend on the relationship with the other person, as well as on the type of situation that is shared (Hess *et al.*, 1995). In line with this, neuroimaging data show that, instead of increasing positive feelings, the presence of an unknown peer during betting decisions increases amygdala activation, possibly as a result of raised alertness (Nawa *et al.*, 2008). Moreover, the interaction mode—cooperation *vs* competition—influences how we differentially process actions of familiar and unfamiliar persons. This has been shown in relation to obtaining gains or losses in a canon shooting game, where unfamiliar others evoked differing responses depending on the interaction mode (de Bruijn *et al.*, 2009). Future work should extend this line of research to specify the determining factors of sharing effects more systematically, investigating the effects of the presence of a range of others, from friends to antagonists, in different interaction modes, and examining the possible role of personality factors and gender in these effects.

In a final note, it is interesting to compare our paradigm with the phenomenon of joint attention as measured by interactive eye-gaze paradigms (Schilbach *et al.* 2006, 2010, 2011). Specifically, as noted, activation in the same reward-related areas was observed when participants’ eye gaze to an object was followed by the eye gaze from another person to the same object (Schilbach *et al.* 2010). In fact, in a certain sense, our paradigm might also be regarded as a kind of ‘joint attention’ paradigm, because it likewise comprises two persons who experience that the other person does or does not draw the attention to the same stimulus. By inviting two actual friends to perform the task simultaneously, our paradigm also fits into the ‘second person’ approach in social neurocience, which aims to develop experimental paradigms for laboratory research that can capture social phenomena of real life better than traditional paradigms (Schilbach *et al.*, 2013). However, the two participants under investigation in our paradigm do not interact in any way, in contrast to the typical joint attention paradigms in which the eye gaze of one partner influences the eye gaze of the other partner. Thus, eye-gaze paradigms inherently investigate externally driven effects depending on the behavior of the partner, while our paradigm is interested in internally driven effects that are independent of the current actual behavior of the partner (although such behavior may psychologically be present in the form of imagination). In terms of the classical definition of social psychology by Gordon Allport as the attempt ‘to understand and explain how the thoughts, feelings and behaviors of individuals are influenced by the actual, imagined, or implied presence of others’ (Allport, 1985), we are focusing on the latter aspect (imagined or implied presence of others), which also in real life is certainly as relevant as the observable behavior of other persons in determining our individual psychological processes. The investigation of such effects based on imagined social connectedness are not in the focus of current models of interpersonal emotion regulation, which refer to effects of open communication behavior (Rimé, 2009; Gable and Reis, 2010; Zaki and Williams, 2013), but in our view would deserve more attention not only in this area but also in social psychology and social neuroscience more generally.

In conclusion, the present study demonstrates how sharing exposure to emotional stimuli with a friend buffers the impact of negative stimuli and enhances the impact of positive stimuli. This was echoed at a neural level by the involvement of the reward circuitry, including VS and OFC. The results add to existing evidence for the importance of social connectedness for our well-being and open up new and interesting avenues of research into mere presence effects (Guerin, 1986; Levine *et al.*, 1993), which have so far focused on performance in cognitive tasks rather than equally important effects in the emotional domain.

## SUPPLEMENTARY DATA

Supplementary data are available at *SCAN* online.

## Conflict of Interest

None declared.

## Supplementary Material

Supplementary Data
