# Supplementary material for: Beautiful friendship: Social sharing of emotions improves subjective feelings and activates the neural reward circuitry
Source: Soc Cogn Affect Neurosci. 2014 Oct 8;10(6):801–8. doi: 10.1093/scan/nsu121 (PMC4448023; doi:10.1093/scan/nsu121)
Supplement: Supplementary Data [file supp_10_6_801__index.html]

Beautiful friendship: Social sharing of emotions improves subjective feelings and activates the neural reward circuitry — Beautiful friendship: Social sharing of emotions improves subjective feelings and activates the neural reward circuitry — Beautiful friendship: Social sharing of emotions improves subjective feelings and activates the neural reward circuitry — Supplementary Data 

# Beautiful friendship: Social sharing of emotions improves subjective feelings and activates the neural reward circuitry

## Supplementary Data

files

**Files in this Data Supplement:**

- Supplementary Data - pdf file
