# Supplementary material for: Beautiful friendship: Social sharing of emotions improves subjective feelings and activates the neural reward circuitry
Source: Soc Cogn Affect Neurosci. 2014 Oct 8;10(6):801–8. doi: 10.1093/scan/nsu121 (PMC4448023; doi:10.1093/scan/nsu121)
Supplement: Supplementary Data [file supp_nsu121_scan-14-081-File002.pdf]

Supplementary Material for:

Beautiful friendship: Social sharing of emotions improves subjective feelings and activates the neural reward circuitry

Ullrich Wagner[1,2]*, Lisa Galli[1]*, Björn H. Schott[1], Andrew Wold[1,3], Job van der Schalk[4], Antony Manstead[4], Klaus Scherer[5] & Henrik Walter[1]

[1] Charité – Universitätsmedizin Berlin, Department of Psychiatry and Psychotherapy, Division of Mind and Brain Research, Berlin, Germany
[2] University of Münster, Department of Psychology, Germany
[3] Berlin School of Mind and Brain, Berlin, Germany
[4] Cardiff University, School of Psychology, Cardiff, UK
[5] Swiss Center for Affective Sciences, Geneva, Switzerland

*equal contribution

Supplementary Results

Arousal

Although our hypothesis referred to the valence dimension of emotion, we also obtained after each picture subjective ratings of arousal as the second primary emotion dimension commonly assessed in emotion research (Lang et al., 2005). Regarding arousal ratings, we did not observe any social sharing effects overall (emotional: M=-0.035, SEM=0.078; t(57)=-0.451, p=0.654; including neutral: M=0.032, SEM=0.072; t(57)=0.456, p=0.650) or when considering positive, negative, and neutral pictures separately (positive: M=-0.060, SEM=0.111; t(57)=-0.548, p=0.586; negative: M=-0.010, SEM=0.079; t(57)=-0.126, p=0.90; neutral: M=0.169, SEM=0.103; t(57)=1.638, p=0.107). (See Supplementary Table S2 for all raw data on subjective ratings of valence and arousal).

Supplementary Tables

(Supplementary Tables S1, S2, S3)

Table S1. Stimulus details. Valence ratings, difference to midpoint of valence scale, and arousal ratings from (Lang et al., 2005). Scales ranging from 1 to 9.

	**Valence**		**Arousal**
		Difference to midpoint	
	Mean (SD)	Mean (SD)	Mean (SD)
negative face	1.67 (1.19)	3.33 (0.08)	6.1 (2.4)
negative nonface	2.5 (1.55)	2.5 (0.34)	5.74 (2.16)
negative inanimate	2.7 (1.58)	2.3 (0.53)	5.5 (2.2)
**negative total**	**2.29 (1.44)**	**2.71 (0.32)**	**5.78 (2.26)**
neutral face	5 (1.28)	0 (0.11)	3.28 (1.94)
neutral nonface	4.98 (1.5)	-0.02 (0.5)	4.29 (2.09)
neutral inanimate	5 (1.01)	0 (0.05)	2.79 (1.91)
**neutral total**	**4.99 (1.26)**	**-0.01 (0.22)**	**3.45 (1.98)**
positive face	8.32 (1.16)	3.32 (0.17)	4.74 (2.58)
positive nonface	7.56 (1.47)	2.56 (0.55)	5.67 (2.31)
positive inanimate	7.37 (1.59)	2.37 (0.21)	4.41 (2.41)
**positive total**	**7.75 (1.41)**	**2.75 (0.31)**	**4.94 (2.44)**

Table S2. Subjective ratings of valence and arousal (scale range 0 -12).

**Valence** Mean (SEM)				
	shared	unshared	raw sharing effect	sharing effect corrected
negative	3.457 (0.146)	3.288 (0.155)	0.168 (0.063)	0.088 (0.056)
positive	8.015 (0.134)	7.921 (0.136)	0.093 (0.071)	0.136 (0.073)
emotional (neg and pos)	5.736 (0.059)	5.604 (0.074)	0.131 (0.045)*	0.112 (0.045)*
neutral	6.196 (0.086)	6.119 (0.082)	0.076 (0.066)	0.064 (0.068)
all	5.888 (0.060)	5.776 (0.069)	0.112 (0.035)*	0.095 (0.037)*

**Arousal** Mean (SEM)				
	shared	unshared	raw sharing effect	sharing effect corrected
negative	6.817 (0.242)	6.865 (0.245)	-0.048 (0.087)	-0.010 (0.079)
positive	5.545 (0.236)	5.500 (0.216)	0.044 (0.094)	-0.060 (0.111)
emotional (neg and pos)	6.181 (0.210)	6.183 (0.203)	-0.001 (0.072)	-0.035 (0.078)
neutral	4.080 (0.242)	3.989 (0.223)	0.090 (0.114)	0.169 (0.103)
all	5.481 (0.208)	5.451 (0.192)	0.029 (0.073)	0.032 (0.072)

* significant effect of social sharing ($p<0.05$) . The corrected sharing effect takes into account that standard ratings for picture sets used in shared vs. unshared experimental conditions cannot be totally matched within subjects and therefore corrects the raw sharing effect of each participant accordingly (see Methods). Overall statistical patterns are the same for raw sharing effects.

Table S3. Regional activation during picture phase. Peak voxels in MNI space, p<0.001 uncorr., k>10.

Region	Hem.	BA	Coordinates peak voxel			T-value	Cluster size
			x	y	Z		
unshared_emo > shared_emo							
Lingual gyus	R	18	12	-76	-5	4.03	40
	L	18	-9	-82	-8	3.8	27
unshared_neu > shared_neu							
Lingual gyrus	R	18	12	-57	1	3.97	231
	R	18	9	-61	-5	3.85	
	R	18	12	-76	-5	3.68	
emo > neu							
Frontopolar cortex	L	10	-3	62	-2	3.87	13
	L	10	0	56	16	3.8	30
	L	10	-9	65	25	3.58	
Anterior cingulate gyrus	L/R	32	0	38	7	3.73	17
Lingual gyrus	R	18	18	-91	-5	3.36	13
Middle temporal gyrus	R	37	54	-70	13	5.95	377
	R	37	54	-58	4	4.34	
Amygdala	L		-21	-4	-14	4.39	157
	R		21	-4	-14	3.94	
Fusiform gyrus	R	37	42	-43	-14	3.79	19
	R	37	30	-73	-5	3.56	21
Precuneus	L/R	23	0	-52	31	4.68	167
	R		3	-49	13	3.92	
Lingual gyrus /Cuneus	L	18	-12	-94	-2	3.88	31
	R	18	18	-91	-5	3.36	13
Middle occipital gyrus	L	19	-48	-76	16	5.47	234
Superior occipital gyrus	R	19	39	-82	31	3.53	
Midbrain	R		3	-16	-14	3.7	11
neg > pos							
Inferior/medial frontal gyrus	R	48	51	23	16	6.34	2365
	R	44	39	5	31	6.2	
	R	46	57	29	16	6.16	
	L	6	-48	2	31	4.73	95
	L	44	-54	14	37	4.01	
	L	48	-45	11	19	3.18	
Superior frontal gyrus	R	10	12	65	28	5.76	45
	R	10	15	68	19	5.35	

	L	32	-6	50	25	4.8	75
	L	9	-6	56	40	4.17	
	L	8	-6	41	55	3.3	
	L	10	-12	62	31	4.26	11
Inferior temporal /	L	37	-42	-58	-8	8.92	6107
inferior occipital gyrus	R	37	45	-67	-8	8.8	
	L	18	-36	-88	4	8.16	
cluster also including:							
Amygdala	R		21	-4	-11	5.16	
	L		-21	-4	-14	4.3	
Hippocampus	L		-27	-10	-11	4.83	
Temporal pole	R	38	48	14	-26	3.74	
Middle temporal gyrus	R	21	51	-4	-17	4.23	61
Brainstem			0	-37	-35	3.93	34
pos > neg							
Medial orbitofrontal gyrus	R	11	9	56	-5	4.77	24
	R	47	39	50	-5	3.79	
	R	46	39	47	7	3.71	
Anterior cingulate gyrus	L	25	-6	35	4	4.98	476
	L	11	-15	44	-2	4.89	
Cingulate gyrus	R	23	9	-13	31	4	59
Hippocampus	R		21	-34	7	4.08	
Precuneus	R	7	9	-73	37	5.26	387
	L	7	-9	-67	31	4.71	
Paracentral lobule	L	4	-6	-19	67	4	153
	R	4	3	-19	67	3.92	
Posterior cingulate gyrus	L	23	-6	-31	25	3.97	
Angular gyrus	R	39	42	-64	43	3.68	10

Table S4. Activation peaks in OFC used for ROI creation.

Source	x	y	z	atlas-type
(Rothkirch, Schmack, Schlagenhauf, & Sterzer, 2012)	0	26	-14	MNI
	30	38	-14	MNI
(Schlagenhauf et al., 2009)	0	45	-12	MNI
	-3	42	-18	MNI
	-6	45	-18	MNI
(Walter et al., 2010)	39	21	-15	MNI
	33	30	-9	MNI
(Diaconescu et al., 2011)	20	20	-24	MNI
(Bunzeck, Dayan, Dolan, & Duzel, 2010)	10	-24	-18	MNI
(Bunzeck, Doeller, Dolan, & Duzel, 2012)	6	48	-20	MNI
	0	46	-26	MNI
	-4	36	-26	MNI
	4	40	-28	MNI
(Liu, Hairston, Schrier, & Fan, 2011)	-46	42	-4	MNI
	-42	52	-6	MNI
(Wagner, Boswell, Kelley, & Heatherton, 2012)	-27	36	-12	MNI
(Diekhof, Falkai, & Gruber, 2011)	21	21	-21	MNI
	-24	27	-21	MNI
	15	18	-18	MNI
	-21	39	-18	MNI
(Bermpohl et al., 2010)	-30	42	-9	MNI
(O'Doherty, Critchley, Deichmann, & Dolan, 2003)	-39	42	-15	MNI
(O'Doherty, Kringelbach, Rolls, Hornak, & Andrews, 2001)	6	42	-24	Tal
	-10	40	-22	Tal
	-4	28	-18	Tal
	36	58	-12	Tal

References

Bermpohl, F., Kahnt, T., Dalanay, U., Hagele, C., Sajonz, B., Wegner, T., . . . Heinz, A. (2010). Altered representation of expected value in the orbitofrontal cortex in mania. *Hum Brain Mapp, 31*(7), 958-969. doi: 10.1002/hbm.20909

Bunzeck, N., Dayan, P., Dolan, R. J., & Duzel, E. (2010). A common mechanism for adaptive scaling of reward and novelty. *Hum Brain Mapp, 31*(9), 1380-1394. doi: 10.1002/hbm.20939

Bunzeck, N., Doeller, C. F., Dolan, R. J., & Duzel, E. (2012). Contextual interaction between novelty and reward processing within the mesolimbic system. *Hum Brain Mapp, 33*(6), 1309-1324. doi: 10.1002/hbm.21288

Diaconescu, A. O., Jensen, J., Wang, H., Willeit, M., Menon, M., Kapur, S., & McIntosh, A. R. (2011). Aberrant Effective Connectivity in Schizophrenia Patients during Appetitive Conditioning. *Front Hum Neurosci, 4*, 239. doi: 10.3389/fnhum.2010.00239

Diekhof, E. K., Falkai, P., & Gruber, O. (2011). The orbitofrontal cortex and its role in the assignment of behavioural significance. *Neuropsychologia, 49*(5), 984-991. doi: 10.1016/j.neuropsychologia.2011.01.032

Liu, X., Hairston, J., Schrier, M., & Fan, J. (2011). Common and distinct networks underlying reward valence and processing stages: a meta-analysis of functional neuroimaging studies. *Neurosci Biobehav Rev, 35*(5), 1219-1236. doi: 10.1016/j.neubiorev.2010.12.012

O'Doherty, J., Critchley, H., Deichmann, R., & Dolan, R. J. (2003). Dissociating valence of outcome from behavioral control in human orbital and ventral prefrontal cortices. *J Neurosci, 23*(21), 7931-7939.

O'Doherty, J., Kringelbach, M. L., Rolls, E. T., Hornak, J., & Andrews, C. (2001). Abstract reward and punishment representations in the human orbitofrontal cortex. *Nat Neurosci, 4*(1), 95-102. doi: 10.1038/82959

Rime, B. (2009). Emotion Elicits the Social Sharing of Emotion: Theory and Empirical Review. *Emotion Review, 1*(1), 60-85. doi: 10.1177/1754073908097189

Rothkirch, M., Schmack, K., Schlagenhauf, F., & Sterzer, P. (2012). Implicit motivational value and salience are processed in distinct areas of orbitofrontal cortex. *Neuroimage, 62*(3), 1717-1725. doi: 10.1016/j.neuroimage.2012.06.016

Schlagenhauf, F., Sterzer, P., Schmack, K., Ballmaier, M., Rapp, M., Wrase, J., . . . Heinz, A. (2009). Reward feedback alterations in unmedicated schizophrenia patients: relevance for delusions. *Biol Psychiatry, 65*(12), 1032-1039. doi: 10.1016/j.biopsych.2008.12.016

Walter, H., Heckers, S., Kassubek, J., Erk, S., Frasch, K., & Abler, B. (2010). Further evidence for aberrant prefrontal salience coding in schizophrenia. *Front Behav Neurosci, 3*, 62. doi: 10.3389/neuro.08.062.2009
